# Machine Learning–Based Text Analysis to Predict Severely Injured Patients in Emergency Medical Dispatch: Model Development and Validation

**DOI:** 10.2196/30210

**Published:** 2022-06-10

**Authors:** Kuan-Chen Chin, Yu-Chia Cheng, Jen-Tang Sun, Chih-Yen Ou, Chun-Hua Hu, Ming-Chi Tsai, Matthew Huei-Ming Ma, Wen-Chu Chiang, Albert Y Chen

**Affiliations:** 1 Department of Emergency Medicine Taipei Hospital Ministry of Health and Welfare New Taipei City Taiwan; 2 Department of Civil Engineering National Taiwan University Taipei City Taiwan; 3 Department of Emergency Medicine Far Eastern Memorial Hospital New Taipei City Taiwan; 4 Emergency Medical Service Division Taipei City Fire Department Taipei City Taiwan; 5 Department of Emergency Medicine National Taiwan University Hospital Taipei City Taiwan; 6 Department of Emergency Medicine National Taiwan University Hospital, Yun-Lin Branch Yunlin County Taiwan

**Keywords:** emergency medical service, emergency medical dispatch, dispatcher, trauma, machine learning, frequency–inverse document frequency, Bernoulli naïve Bayes

## Abstract

**Background:**

Early recognition of severely injured patients in prehospital settings is of paramount importance for timely treatment and transportation of patients to further treatment facilities. The dispatching accuracy has seldom been addressed in previous studies.

**Objective:**

In this study, we aimed to build a machine learning–based model through text mining of emergency calls for the automated identification of severely injured patients after a road accident.

**Methods:**

Audio recordings of road accidents in Taipei City, Taiwan, in 2018 were obtained and randomly sampled. Data on call transfers or non-Mandarin speeches were excluded. To predict cases of severe trauma identified on-site by emergency medical technicians, all included cases were evaluated by both humans (6 dispatchers) and a machine learning model, that is, a prehospital-activated major trauma (PAMT) model. The PAMT model was developed using term frequency–inverse document frequency, rule-based classification, and a Bernoulli naïve Bayes classifier. Repeated random subsampling cross-validation was applied to evaluate the robustness of the model. The prediction performance of dispatchers and the PAMT model, in severe cases, was compared. Performance was indicated by sensitivity, specificity, positive predictive value, negative predictive value, and accuracy.

**Results:**

Although the mean sensitivity and negative predictive value obtained by the PAMT model were higher than those of dispatchers, they obtained higher mean specificity, positive predictive value, and accuracy. The mean accuracy of the PAMT model, from certainty level 0 (lowest certainty) to level 6 (highest certainty), was higher except for levels 5 and 6. The overall performances of the dispatchers and the PAMT model were similar; however, the PAMT model had higher accuracy in cases where the dispatchers were less certain of their judgments.

**Conclusions:**

A machine learning–based model, called the PAMT model, was developed to predict severe road accident trauma. The results of our study suggest that the accuracy of the PAMT model is not superior to that of the participating dispatchers; however, it may assist dispatchers when they lack confidence while making a judgment.

## Introduction

### Background

Trauma is a leading cause of accidental death globally. According to the World Health Organization, injuries contribute to >5 million deaths each year. Road traffic accidents accounted for most injuries and were the ninth leading cause of death in 2012 [1]. Severe trauma is a time-sensitive emergency condition. Prompt transport is beneficial for patients with neurotrauma and penetrating injuries with unstable hemodynamic features [2]. Delays in transportation are associated with poor functional outcome [3].

Prehospital triage allows severely ill patients to receive appropriate time-sensitive management. For cardiac arrest and stroke victims, dispatchers can obtain critical information on the phone, such as the patient’s level of consciousness, breath patterns, or prehospital stroke scales [4,5]. However, no standardized questions have been designed for dispatchers when they encounter severe trauma. Only a few studies on helicopter emergency medical services have addressed the accuracy of dispatch for trauma victims [6]. Current trauma scales for predicting severity require either physiological or anatomical assessments [7]. Therefore, a victim’s condition cannot be identified or evaluated until the first batch of emergency medical technicians (EMTs) arrives at the scene.

### Motivation

Content analysis has been conducted on emergency calls to discover the factors that affect dispatch and have the potential to assist prehospital triage [8,9]. Specifically, text classification has demonstrated the effectiveness of classifying events recorded during phone calls [10]. In addition, natural language processing has been used in emergency medicine. Text mining techniques have been used to predict the triage level, length of stay, disposition, and mortality in emergency department patients [11-16]. A textual analysis–based machine learning framework was developed to assist dispatchers during the prehospital phase in out-of-hospital cardiac arrest (OHCA) recognition; this framework has been commercialized [17-20]. These techniques make it possible to stratify the risk to patients when structured questions are unavailable, similar to the assessment of trauma patients over the phone.

The classic process of text classification includes text preprocessing, feature extraction, and classifier construction. Text preprocessing aims to remove noise and effectively retrieve information through text cleaning and organization [21]. Common feature extraction approaches can be loosely divided into two domains: word frequency and semantics [22,23]. Machine and deep learning models, such as k-nearest neighbors, decision trees, support vector machines, multilayer perceptron classifiers, and naïve Bayes, are widely used as classifiers [24-28].

### Aim

We hypothesized that severe trauma cases could be recognized based on the content of communication between callers and call takers during emergency calls. The main research question and objective of this study was to develop a machine learning–based model through text mining of emergency calls to automatically identify severely injured patients in road accidents. We focused on road accidents instead of all trauma cases because they are the major cause of trauma, and compared with other types of injuries, the content of emergency calls for road accidents is homogeneous. As there are no suitable previous studies for comparison, our second objective was to compare the results of the model with 6 participating dispatchers’ judgment.

## Methods

### Study Design and Setting

This paper describes a cross-sectional study on identifying severely injured patients in road accidents by analyzing Mandarin text of emergency calls using machine learning. The results were compared with those of human judgment. We defined severely injured patients as those who fit the major trauma criteria of the EMT trauma triage protocol, that is, prehospital-activated major trauma (PAMT).

#### Data Acquisition

**Figure 1:**
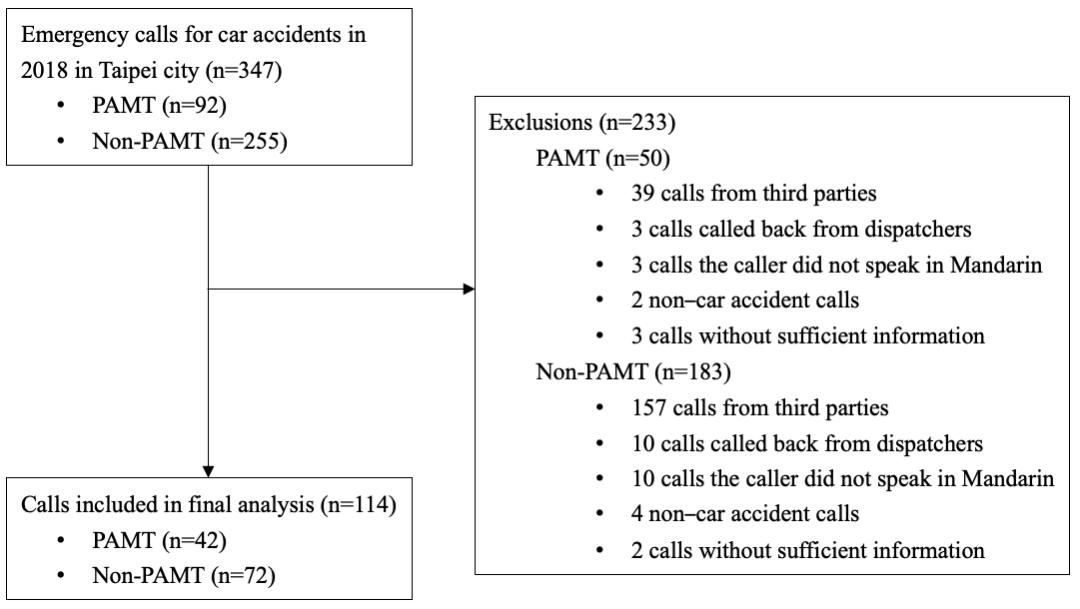
Data acquisition and study design. PAMT: prehospital-activated major trauma.

Data were obtained from the Taipei Trauma Registry, which is a database of trauma accident information from 8 out of 18 hospitals with first aid capabilities. A random sample of one-fourth of the total cases considered as PAMT in 2018 was retrieved. After excluding cases without complete information, 92 PAMT patients (92 of 377 registered cases) were enrolled. As control cases, 3 consecutive non-PAMT road accident calls were matched with each PAMT on the same day from the dispatch system. If the number of non-PAMT cases to be matched on a given day was insufficient, only 1 or 2 calls were included. A total of 92 PAMT calls and 255 non-PAMT calls were considered in this study. The exclusion criteria were as follows: the caller was not by the side of the victim, the caller did not speak Mandarin, the accident was not vehicle-related, and the calls did not provide sufficient information. The final data for analysis included 114 cases in total, which comprised 42 PAMT and 72 non-PAMT cases (Figure 1).

#### Ethics Approval

This study was approved by the institutional review board of the National Taiwan University Hospital (case number 201902043RINB).

### Model Development

**Figure 2:**
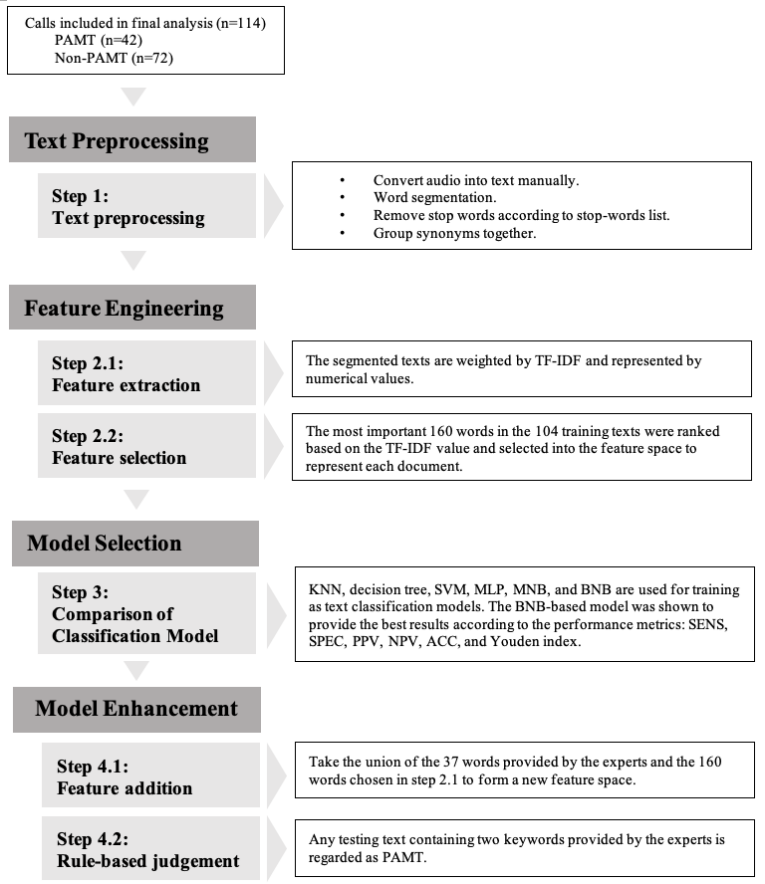
Model development. PAMT: prehospital activated major trauma; TF-IDF: term frequency–inverse document frequency; KNN: k-nearest neighbors; SVM: support vector machine; MNB: multinomial naïve Bayes; BNB: Bernoulli naïve Bayes; MLP: multilayer perceptron; SENS: sensitivity; SPEC: specificity; PPV: positive predictive value; NPV: negative predictive value; ACC: accuracy. Repeated random subsampling-cross validation (RRS-CV) for 100 times were performed in the step of model enhancement. All training data include 39 PAMT and 65 non-PAMT cases; testing data included 3 PAMT and 7 non-PAMT cases.

As shown in Figure 2, formal model development comprises four steps: (1) text preprocessing, (2) feature engineering, (3) model classification, and (4) model enhancement, which was conducted to improve model performance.

#### Text Preprocessing (Step 1)

The purpose of text preprocessing is to organize the data such that useful information can be retrieved. This process includes word segmentation, stop word removal, and synonym grouping (Figure 2). First, each emergency call was manually converted into a text form. The continuous text string was then segmented into words, which were the shortest units of meaning, consisting of at least one character. Segmentation was performed using the Chinese word segmentation system developed by the Institute of Information Science and the Institute of Linguistics of Academia Sinica [29,30]. To eliminate segmentation errors caused by ambiguous Chinese compound words, a dictionary of special terms with specific weights was manually constructed based on experience and trial and error. The segmentation system refers to the weight required to force certain words to merge or separate. Subsequently, stop words were removed to remove insignificant words, such as conjunctions, pronouns, and articles. Then, synonyms were grouped and regarded as the same word, potentially reducing the model overfitting to specific words, thus providing a means for bias-variance control. From >27,000 characters in the original 114 texts, approximately 7000 different word meanings were identified.

#### Feature Engineering (Step 2)

In feature engineering, the segmented words were transformed into a machine-readable format by feature extraction (step 2.1, Figure 2). As emergency calls are often short, and conversations are urgent, important words are frequently mentioned (Multimedia Appendix 1). Thus, we used term frequency–inverse document frequency (TF-IDF) to weigh each word. The TF-IDF calculation consists of two sections: TF and IDF (Multimedia Appendix 2). TF illustrates the word frequency, whereas IDF explains the rarity of words appearing in the entire document. A higher frequency of occurrence of a word in one specific text indicates its importance. In contrast, a higher frequency of occurrence of the word in the entire body of texts lowers its importance. By considering these 2 frequencies simultaneously, we ranked all words by importance to conduct feature selection (step 2.2, Figure 2). The most important 160 out of 7000 words were chosen based on the experiments. The selected features were placed in a feature space to reduce the number of dimensions and to make the results more explanatory. The feature space included the selected features used to develop the model.

#### Model Selection (Step 3)

For model selection, we evaluated several commonly used machine learning models for text classification, including k-nearest neighbors, decision tree, support vector machine, multilayer perceptron, multinomial naïve Bayes, and Bernoulli naïve Bayes (BNB). Repeated random subsampling cross-validation (RRS-CV) was conducted 100 times to avoid overfitting and to obtain more stable and reliable classification results. RRS-CV splits samples in a randomized and repeated manner without replacement. The performance of the different models used for comparison was the average of 100 RRS-CV scores. According to Table 1, among these, the BNB-based model achieved the best results. The BNB classifier, which is a supervised learning model, is based on Bayes’ theorem. It assumes that each input variable is independent of the other variables. According to the BNB equation in Multimedia Appendix 2, the calculation concentrates on binary information of whether the word appears in a document. The Boolean expression of the selected features forms the feature vector for each document. The category estimation of a document depends on the maximum a posteriori of each class *k*, which consists of the likelihood of the document being given by class *k* and its prior probability. The category with the highest maximum a posteriori labeled the classified documents. To avoid a zero-probability situation, Laplace smoothing was used to set the additive smoothing parameter to one. Consequently, no hyperparameter tuning was required for BNB. Compared with other text classification models, the BNB model has the advantages of simplicity, efficient computational speed, and ability to achieve a high level of accuracy without hyperparameter tuning. Furthermore, this model is suitable for processing small-scale data and short texts [31,32]. The results and hyperparameter tuning of other models are presented in Multimedia Appendix 3.

**Table 1:** Comparison of machine learning models.

Model	SENS^a^ (%)	SPEC^b^ (%)	PPV^c^ (%)	NPV^d^ (%)	ACC^e^ (%)	Youden index
KNN^f^	18.7	89.0	32.6	72.1	67.9	0.077
Decision tree	32.7	76.0	35.9	72.9	63.0	0.087
SVM^g^	55.7	74.0	49.3	80.3	68.5	0.297
MNB^h^	19.0	96.1	42.2	73.8	73.0	0.151
BNB^i^	*53.0* ^j^	*86.7* ^j^	*67.0* ^j^	*81.6* ^j^	*76.6* ^j^	*0.397* ^j^
MLP^k^	53.7	79.0	55.6	80.6	71.4	0.327

^a^SENS: sensitivity.

^b^SPEC: specificity.

^c^PPV: positive predictive value.

^d^NPV: negative predictive value.

^e^ACC: accuracy.

^f^KNN: k-nearest neighbors.

^g^SVM: support vector machine.

^h^MNB: multinomial naïve Bayes.

^i^BNB: Bernoulli naïve Bayes.

^j^BNB-based model achieved the best ACC and Youden index.

^k^MLP: multilayer perceptron.

**Figure 3:**
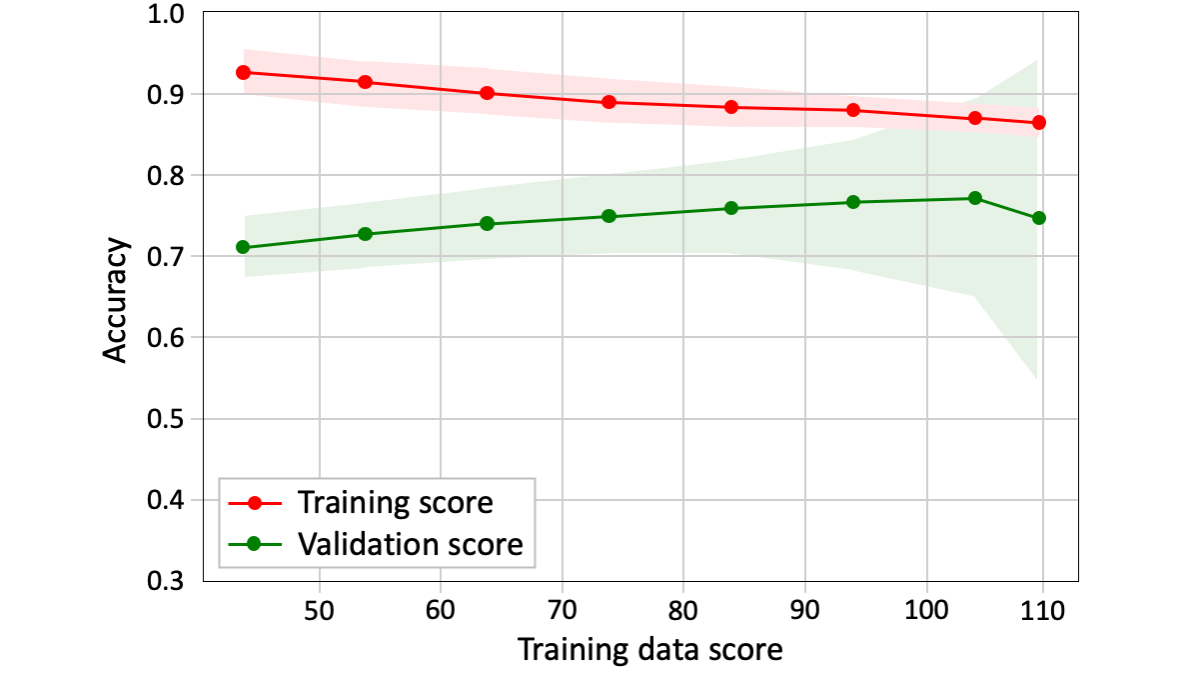
Learning curve of the Bernoulli naïve Bayes.

**Figure 4:**
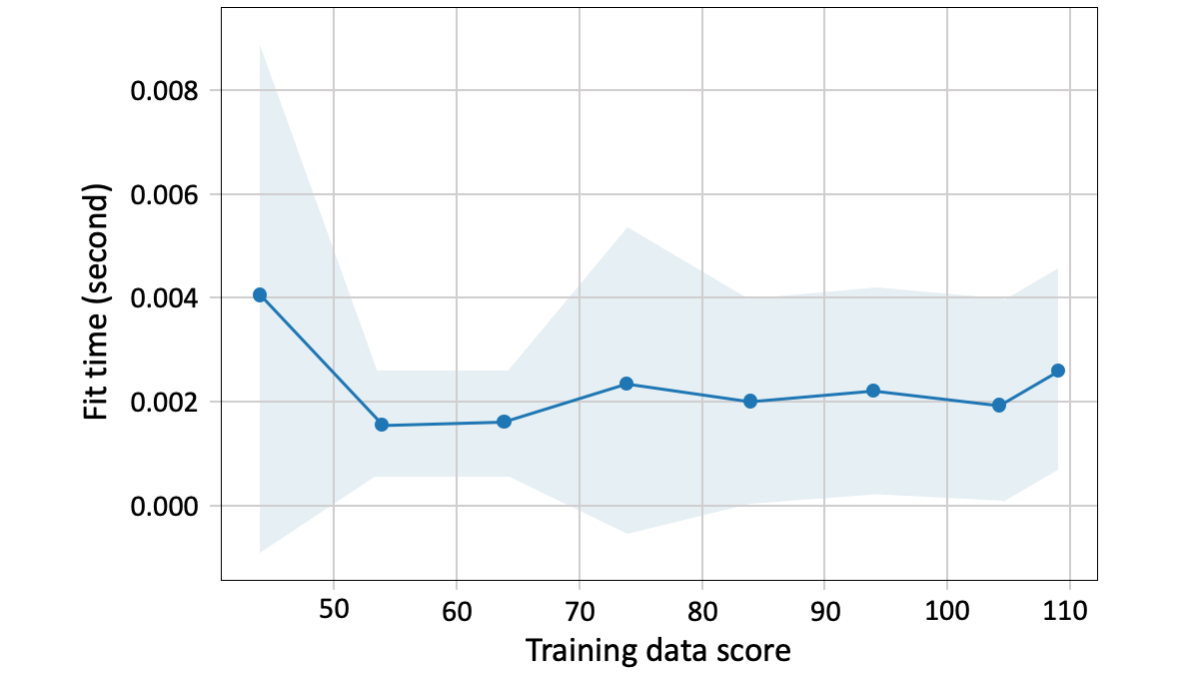
Scalability of the Bernoulli naïve Bayes.

**Figure 5:**
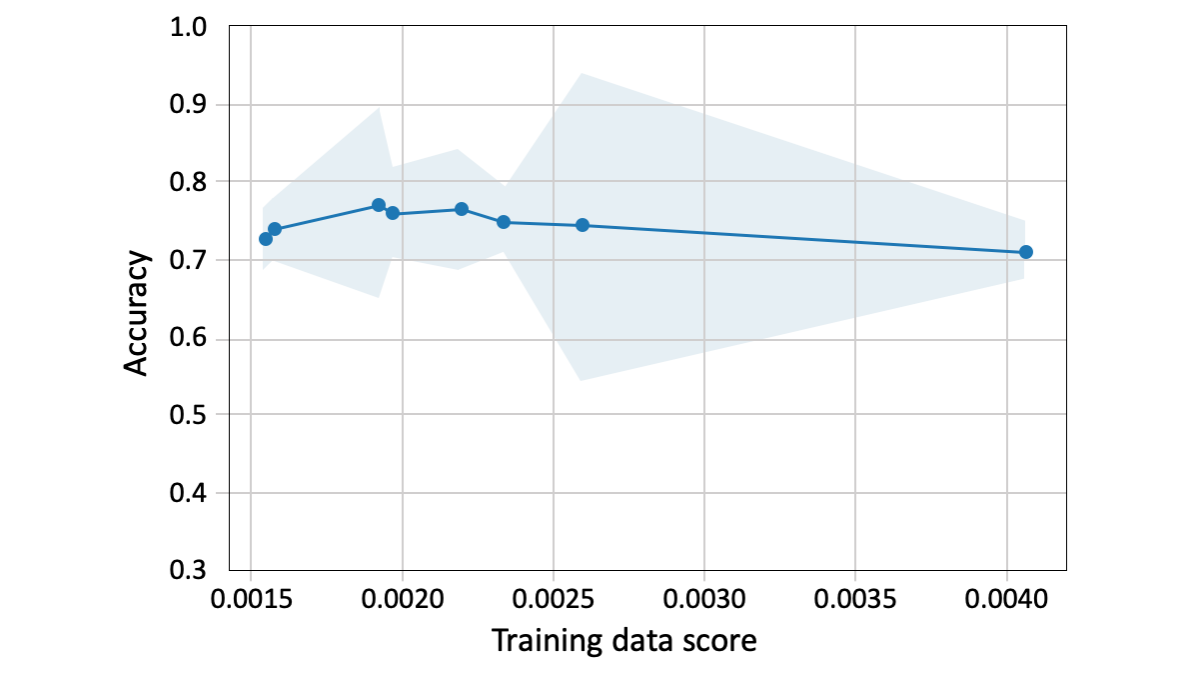
Performance of the Bernoulli naïve Bayes.

For the split of training and validation data, we set a fixed ratio of PAMT to non-PAMT cases in the validation data. As shown in Figure 3, when the amount of training data becomes larger than that of the validation data, the training score gradually decreases and the validation score increases. The 2 lines were closest when the training and validation data sizes were 104 and 10, respectively. The convergence illustrates that, at this number of training samples, adding more training data does not significantly improve the classification performance. Therefore, for all text classification models, 104 texts were randomly selected as training data and the remaining 10 texts were used as validation data (Figure 2). The training data included 39 PAMT and 65 non-PAMT cases, and the validation data included 3 PAMT and 7 non-PAMT cases. The ground truth of model classification is the on-scene judgment of the EMT, which is presented in the form of binary labels. Figure 4 shows the scalability of the BNB-based model. As the training data increased, the model-fitting time fluctuated moderately around 0.002 seconds but significantly increased when the training data size was >104. In addition, 104 training data points with 10 validation data points had the highest validation score and the third shortest model-fitting time (Figure 5).

#### Model Enhancement (Step 4)

To optimize the final performance of our model, we enhanced the BNB-based models using feature addition (step 4.1) and rule-based judgment (step 4.2). In feature addition, we gathered 37 keywords provided by the experts and combined them with the 160 words chosen in step 2.2 to form a new feature space (Figure 2). The experts included 6 participating dispatchers and 2 emergency physicians. After they had listened to the 114 audio recordings, they were asked, “Which keyword in an emergency call indicates whether a patient is a PAMT or non-PAMT patient?” They then provided keywords based on their personal experience. The 37 keywords were expected to expand the important feature set, which may be limited by the small amount of data. The feature space created by the union of 160 and 37 words was used to develop enhanced models. Although important features must be included, their contribution to the classification may be small if their frequencies are not significant. Therefore, a rule-based judgment (step 4.2) was designed to highlight the importance of the 37 suggested keywords. Specifically, any text used in the validation that contained at least 2 of the 37 words provided by the experts was classified as PAMT. Texts that did not fit this rule were further examined by a BNB classifier (Figure 2).

The enhanced BNB-based model was compared with various derivative models based on combinations of different steps. The 4 derivative versions of the BNB-based model are presented in Table 2. Model A comprised manually selected features and rule-based judgment. Model B was a classical text classification model that included TF-IDF feature extraction and selection with BNB classification. Model C comprised feature engineering steps and manual feature addition with BNB classification. Finally, we named the best version as the PAMT model. It comprises steps 1 to 4.2, including text preprocessing, feature engineering, model classification, and both model enhancement approaches.

**Table 2:** BNB-based models of different combinations of steps.

Model	Performance							Steps included^a^					BNB^b^ classification
	SENS^c^ (%)	SPEC^d^ (%)	PPV^e^ (%)	NPV^f^ (%)	ACC^g^ (%)	Youden index	1		2	4.1	4.2		
Model A	54.7	82.1	56.8	80.9	73.9	0.368	✓			✓	✓		
Model B	53.0	86.7	67.0	81.6	76.6	0.397	✓		✓			✓	
Model C	54.0	87.3	67.8	82.1	77.3	0.413	✓		✓	✓		✓	
PAMT^h^ model	68.0	78.0	60.6	85.8	75.0	0.460	✓		✓	✓	✓	✓	

^a^Step 1, text preprocessing; step 2, term frequency–inverse document frequency feature extraction and selection; step 4.1, manual feature addition; step 4.2, rule-based judgment.

^b^BNB: Bernoulli naïve Bayes.

^c^SENS: sensitivity.

^d^SPEC: specificity.

^e^PPV: positive predictive value.

^f^NPV: negative predictive value.

^g^ACC: accuracy.

^h^PAMT: prehospital-activated major trauma.

### Human Participants

For a reference comparison with the PAMT model, we conducted a survey to collect severe trauma judgments from 6 volunteer dispatchers. They were from the fire departments of Taipei City and New Taipei City (Table 3). The participants were asked to listen to 114 road accident audio clips. As we focused on text analysis, the participants were not allowed to receive any information other than the text. Therefore, the audio clips were transcribed into a computer-synthesized voice using a text-to-speech tool. The audio clips were played randomly in both female and male voices. In this way, the tone, speed, and emotions of the speech were neutralized. While listening to the clips, each participant classified the cases as PAMT or non-PAMT depending on their personal experience and intuition. They also shared information regarding their certainty (certain or uncertain) in each case.

**Table 3:** Profiles of the participating dispatchers.

Participant	Sex	Age (years), range	Service city	EMT^a^ experience (year)	Dispatch experience (year)
A	Male	30-39	New Taipei City	13	6
B	Female	40-49	New Taipei City	10	2
C	Male	30-39	New Taipei City	14	1
D	Male	30-39	New Taipei City	10	1
E	Male	30-39	Taipei City	10	4
F	Male	30-39	Taipei City	9	4

^a^EMT: emergency medicine technician.

### Data Analysis

The analysis determined the accuracy, positive predictive value, negative predictive value, sensitivity, and specificity of the PAMT model prediction and average judgments of the participants [33,34].

Accuracy refers to the proportion of correctly predicted PAMT and non-PAMT cases. The proportion of cases with true-predicted PAMT and non-PAMT results can be presented as positive predictive value and negative predictive value, respectively. sensitivity and specificity represent the ability of a classification system to correctly identify PAMT and non-PAMT cases, respectively. The Youden index was calculated using different models and can be expressed as the sum of sensitivity and specificity minus 1.

All 114 cases were categorized into certainty levels from 0 to 6, depending on how many participants regarded a case as *certain*. For example, a case with certainty level 4 indicated that 4 participants were certain of their judgment, whereas the other two were not. The accuracy was also calculated for different certainty levels.

Data management and statistical analyses were performed using Python (Python Software Foundation) and Excel (Microsoft Corporation).

## Results

### Sample

In total, 114 patients were included in the final analysis. The transcribed texts ranged from 84 to 652 characters, with a mean of 241.4 (SD 106.7) characters; the mean character count of PAMT cases was greater than that of non-PAMT cases (266, SD 102 vs 227, SD 107). The transcribed computer-synthesized audio ranged from 24 to 145 seconds in length, with a mean of 58.9 (SD 24.5) seconds, and the mean call length of PAMT cases was longer than that of non-PAMT (64, SD 24 vs 54, SD 24 seconds) cases (Multimedia Appendix 4).

### Outcome Data

In this study, the machine learning model was trained on a random sample of 104 cases and validated on the remaining 10 cases. RRS-CV was conducted 100 times to obtain greater unbiased validation results; moreover, no external data were used to test the performance of the trained models. According to Table 1, BNB outperformed the other models because it had the highest overall metrics: accuracy (76.6%) and Youden index (0.397). The mean sensitivity, specificity, positive predictive value, and negative predictive value for BNB were 53.0%, 86.7%, 67.0%, and 81.6%, respectively. As there was still room for improvement, model enhancement was performed based on BNB to increase the performance. The enhanced BNB-based model, known as the PAMT model, exhibited the best performance. Its Youden index was 0.460, and it achieved a mean sensitivity, specificity, positive predictive value, negative predictive value, and accuracy of 68.0%, 78.0%, 60.6%, 85.8%, and 75.0%, respectively (Table 2). The performance of model C, which was only enhanced by adding the features provided by the 6 volunteer dispatchers, was ranked after the PAMT model. The mean sensitivity, specificity, positive predictive value, negative predictive value, accuracy, and Youden index of model C were 54.0%, 87.3%, 67.8%, 82.1%, 77.3%, and 0.413, respectively. Model A contained only the features provided by the experts and was classified based on rule-based judgment. It achieved the worst results (sensitivity 54.7%; specificity 82.1%; positive predictive value 56.8%; negative predictive value 80.9%; accuracy 73.9%; Youden index 0.368).

**Figure 6:**
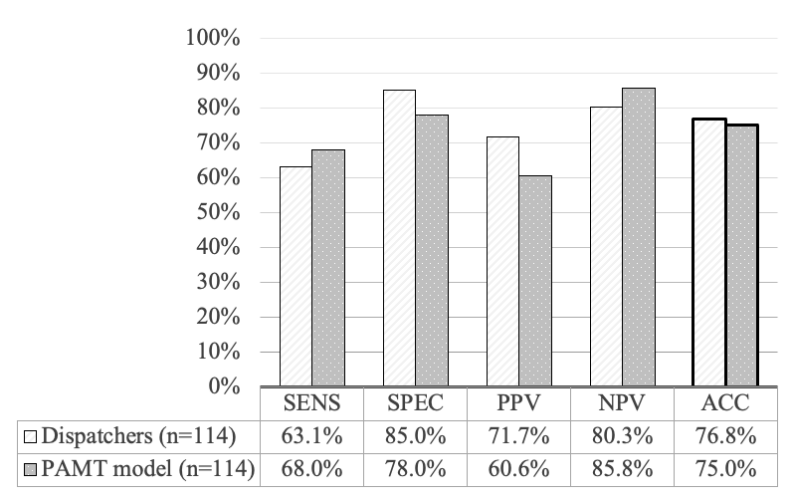
Overall performance of participating dispatchers versus prehospital-activated major trauma (PAMT) model. ACC: accuracy; NPV: negative predictive value; PAMT: prehospital activated major trauma; PPV: positive predictive value; SENS: sensitivity; SPEC: specificity.

In contrast, the mean sensitivity, specificity, positive predictive value, negative predictive value, and accuracy of the 6 participants were 63.1%, 85.0%, 71.7%, 80.3%, and 76.8%, respectively (Multimedia Appendix 5). The PAMT model with the best performance had a higher sensitivity and negative predictive value but a lower specificity, positive predictive value, and accuracy than the participants. Overall, the PAMT model did not surpass the performance of the participating dispatchers (Figure 6).

**Figure 7:**
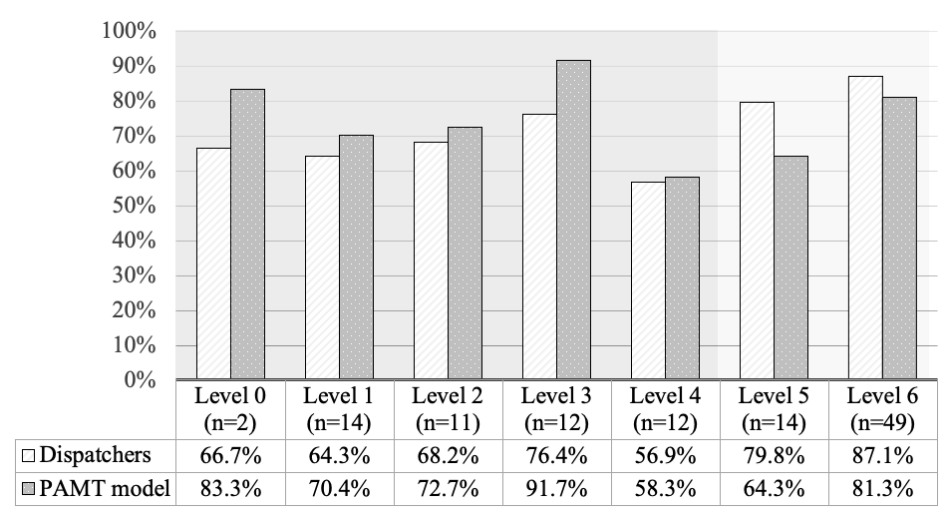
Accuracy of predicting prehospital-activated major trauma (PAMT) by participating dispatchers and PAMT model over different certainty levels.

In the subgroup analysis, as shown in Figure 7, the mean accuracy of the participants at certainty levels from 0 to 6 was 66.7%, 64.3%, 68.2%, 76.4%, 56.9%, 79.8%, and 87.1%. The mean accuracy of the PAMT model at certainty levels from 0 to 6 was 83.3%, 70.4%, 72.7%, 91.7%, 58.3%, 64.3%, and 81.3%. After all cases were categorized based on different certainty levels, the accuracy of the participants for levels 0 to 6 generally increased, except for level 4, whereas the accuracy of the PAMT model did not show such a linear pattern. The results of the PAMT model did not display a clear trend; that is, they were affected by the certainty level because the BNB model classified cases according to the feature distribution. If we define levels 5 and 6 as *certain cases* and levels 0 to 4 as *uncertain cases*, we can observe that, although the accuracy of the PAMT model was lower than that of the participants in *certain* cases (77.52% vs 85.48%), it was greater than the accuracy of the participants in *uncertain* cases (73.57% vs 66.34%; Figure 7).

## Discussion

### Principal Findings

Our study makes 3 major contributions to the field. First, this is the primary study to use a machine learning–based model to identify severely injured patients during the dispatch phase. Second, the overall performance of the model was similar to that of human dispatchers (Figure 6). Third, the model produced favorable results for cases in which dispatchers were uncertain (Figure 7).

With no suitable previous studies as a reference, we enrolled 6 volunteer dispatchers in our study. Their judgment was regarded as a reference for comparison with the models. Although such a small sample size cannot represent all dispatchers, we were still able to observe heterogeneity in human performance. As shown in Multimedia Appendix 5, three participants (A, B, and E) had a high specificity and low sensitivity, whereas the other three (C, D, and F) had more balanced figures between specificity and sensitivity. We can speculate that different experiences may affect judgment, and that the policy each participant chose, either aggressive or conservative, also made a difference. With the assistance of the proposed model, which is more stable and adjustable, it is possible to narrow the range of human discrepancies and decrease the uncertainty.

The proposed machine learning models are text classification models. As important words were repeatedly mentioned in often short and intermittent emergency calls (Multimedia Appendix 1), the frequency-based feature extraction method, TF-IDF, demonstrated the ability to select representative words in severe trauma calls. In addition, feature correlation analysis was performed for these words (Multimedia Appendix 6). Features with high correlation coefficients were words that frequently appeared together in Mandarin, or in the question required to be asked during a call. In contrast, low correlation words indicated that they appeared independently. Despite varying degrees of correlation, all the selected features are meaningful and have the potential to be keywords for judging PAMT. Therefore, the occurrence of these words was the main input for the machine learning models. Furthermore, we analyzed the length of the texts and the accuracy between the PAMT and participants. As each text was represented by a feature vector formed by word occurrences, the original length may be one of the factors affecting accuracy. Multimedia Appendix 7 presents further results.

To explore why machine learning performed better in classifying uncertain cases, we compared the words suggested by experts and the words selected by the model. Of the 37 words provided by the experts, 23 were regarded as keywords specifically for PAMT. In Multimedia Appendix 8, we compare these 23 words and the top 23 decisive words selected by the model that were most likely to occur in the PAMT texts. In the left column, most words are aggregated in the “Patient status” and “Patient basic information” categories; few are in the “Geographic information” and “Auxiliary words and other information” categories. In contrast, the words in the right column are grouped not only in the “Patient status” category but also in “Geographic information” and “Auxiliary words and other information.” This phenomenon shows that the participants focused more on the situations and injury mechanisms of the patients, whereas the proposed model was able to capture other information such as the location of an accident or wording in a conversation. In uncertain cases, there may be fewer obvious keywords for PAMT, which is possibly why the proposed model is more helpful.

In addition to the PAMT model, we tried different feature combinations and classification approaches to develop three other models. Models A, B, and C refer to manual feature addition with rule-based judgment, TF-IDF feature engineering with BNB classification, and TF-IDF feature engineering plus manual feature addition with BNB classification, respectively (Table 2). The PAMT model consisted of steps 2.1 to 4.2. It is important to consider sensitivity and specificity while developing a triage tool; therefore, we chose the model with the highest Youden index as our final model, which was the PAMT model. The sensitivity of the PAMT model was also the highest, making it suitable for use as a triage tool.

Our results demonstrate that it is necessary to combine machine learning (steps 2.1 and 2.2) and human experience (steps 4.1 and 4.2) to develop a prehospital dispatching triage tool (Table 2). A purely manual model using the features provided by experts with rule-based judgment, such as model A, or a classical machine learning–based text classification model, such as model B, did not perform sufficiently well. Although the features of model C are composed of the TF-IDF selection and are provided by experts, without rule-based judgment to increase the importance of these keywords, it failed to outperform the PAMT model. Rule-based judgment makes the added feature of experts suggesting words more significant in classification, which is a complement of limited data. Although the best classification performance of the BNB model indicates that the occurrence of words in a call is key to identifying PAMT cases, there is currently no machine learning model that can completely replace human dispatchers.

### Comparison With Prior Work

Abundant research has been conducted on field triage and prognosis prediction using prehospital data for the early recognition of severely injured trauma patients. However, the dispatching accuracy has seldom been addressed in previous studies [35,36]. The predictors used in these studies, either physiological data or injury mechanisms, were difficult to acquire through telephone calls. In the few studies regarding the accuracy of dispatching, most dealt with helicopter emergency medical services dispatching [6,37-40]. In another study, all trauma emergency calls were included and compared between clinicians and nonclinicians in a prehospital critical care team in Scotland [41]. The sensitivity of the two groups, in the study, for identifying major trauma (injury severity score>15) were 0.112 and 0.259 and the specificity was 0.998 and 0.995. Our model had a significantly higher sensitivity and lower specificity. However, the results varied as the gold standards differed. We chose the judgment of the on-scene EMT as the gold standard as it represents comprehensive prehospital information, whereas injury severity score is prognostic data that can only be obtained in the hospital. Moreover, a higher sensitivity, which avoids undertriage, allows us to apply the model as an early triage tool that can determine the priority of dispatching based on patient severity.

For a machine learning dispatch support system, a commercialized model for the recognition of OHCA through dispatching was proposed [17]. The model consists of an automatic speech recognition and textual analysis. In 2 retrospective studies conducted in Denmark and Sweden, positive results were reported in terms of both accuracy and time [18,19]. In a randomized controlled trial in Denmark, the performance of this model surpassed human recognition; however, no significant improvements were found in dispatchers’ ability to recognize OHCA with model assistance [20]. Although there are numerous differences in recognizing OHCA and severe trauma, this model also uses machine learning–based text analysis. Another machine learning–based voice analysis model was proposed to recognize the emotional state of OHCA callers [42]. Although the goal differed from our approach, the study also had a small sample size, and the data source was the audio of emergency calls. It is reasonable to expect that the performance of future models may improve with a combination of semantic and emotional analyses.

### Limitations

Our study had several limitations. First, human intervention is required for text preprocessing. Conversations in the recordings were often in fragments without a complete grammatical structure and contained specific terms. A customized dictionary for word segmentation, stop word removal, and synonym grouping must be constructed according to the specific medical domain, regional features, and culture. Although there are references, applying these procedures requires researchers to fully comprehend phone conversations [29]. We assumed that everyone who understood the conversation would process the audio and text materials in the same way; otherwise, the features we used in the later steps would be different. This limitation can be overcome by replacing this step with an automatic program [10]. Second, the dispatchers listened to 114 audio clips before providing the keywords. Although they did not know the answers and were asked to provide their opinions based on their experience, they might have chosen words from the audio that they had just heard. Third, owing to strict protocols and the administration’s concern for this novel study concept, the recordings of the emergency calls were not allowed to be copied, and only a limited number of crews were permitted to access them over a short period. Given that the preprocessing steps were labor intensive, we could not enroll a large sample size. To compensate for this shortage, we randomly sampled the PAMTs during the entire year of 2018 in the Taipei Trauma Registry. For text classification, the main factors that affect the results may not be determined by data size [43]. Another innovative study with a small amount of data contributed to specific fields [42]. This study serves as a proof of concept and aims to reveal the potential of this methodology for target applications. Nevertheless, some advanced text classification models, such as deep learning models with semantic feature extraction methods [44], may be limited by the size and characteristics of the data. Therefore, research should be conducted on a larger scale with more participants and integrated data to develop a more mature model for actual deployment.

### Conclusions

The results of our study suggest that the applied machine learning model is not superior to dispatchers in identifying road accident calls in severe trauma cases; however, the model can assist dispatchers when they lack confidence in the judgment of the calls. A study conducted on a larger scale is required for further model development and validation.

## Appendix

Multimedia Appendix 1 Example texts.

Multimedia Appendix 2 Equations and Python script used in the model.

Multimedia Appendix 3 Comparison of machine learning models.

Multimedia Appendix 4 Descriptive statistics for audio and text files.

Multimedia Appendix 5 Profiles and predictive performances of the participating dispatchers.

Multimedia Appendix 6 Feature correlation analysis.

Multimedia Appendix 7 Relation of the length of text and accuracy.

Multimedia Appendix 8 Representative keywords for prehospital-activated major trauma (PAMT) chosen by experts and the PAMT model.

## Abbreviations

BNB: Bernoulli naïve Bayes
EMT: emergency medical technician
OHCA: out-of-hospital cardiac arrest
PAMT: prehospital-activated major trauma
RRS-CV: repeated random subsampling cross-validation
TF-IDF: term frequency–inverse document frequency
