# Supplementary material for: Machine Learning–Based Text Analysis to Predict Severely Injured Patients in Emergency Medical Dispatch: Model Development and Validation
Source: J Med Internet Res. 2022 Jun 10;24(6):e30210. doi: 10.2196/30210 (PMC9233260; doi:10.2196/30210)
Supplement: Multimedia Appendix 1 [file jmir_v24i6e30210_app1.docx]

Appendix 1. Example texts

The example texts are selected based on the sample with the highest accuracy of the RRS-CV (Repeated Random Subsampling-Cross Validation). They are the original conversations of the non-PAMT and PAMT cases, which did not undergo any synonym substitution and stop word removal.

- Non-PAMT case

| This is fire department.  It’s fire or medical?  Just right here, Jian-Guo, the, Culture University Jian-Guo South Road.  Jian-Guo and what Road?  Jian-Guo and that, Jian-Guo, and that, He-Ping East Road intersection.  He-Ping East Road, right?  Yes yes yes yes.  How many people are hurt?  Car accident. Two two motorcycles involved. The patient is trapped under one of the motorcycle.  Is he awake? Is he breathing normally?  He is awake but under the motorcycle.  OK. I’m sending an ambulance there. Are there two motorcycles?  Yes yes.  Two motorcycle accident with one, one patient, is that right?  No, they are both hurt. One of them are trapped under the motorcycle.  Please hurry.  Madam, the ambulance is on the way.  Are you sure that they are both awake?  Yes, awake, but…  Two of them…the patient who can walk…Is he going to the hospital?  I…I…because there are two motorcycles with one couple and one of is trapped…He supposes…I’m not sure if the couple is going to… but now…  Is it possible you go to ask the couple, are they going to the hospital? This couple, this, the one who trapped under the vehicle. OK we have already sent an ambulance. Is the rider of another motorcycle going to the hospital?  He is just fine right now.  OK, then. Sending just one ambulance first.  Please help to guide the ambulance.  Yes yes thanks.  Sure.  Bye-bye. |
| --- |

- PAMT case

| 911 Fire Department. Ambulance or  Hello, hello.  fire truck?  A car accident happened here.  What’s the address of the accident?  Well, I’m at the Shin-Kong Mitsukoshi, on Zhong-Xiao East Road.  Which section of Zhong-Xiao East Road? Don’t panic. Go check the house number or the intersection of the roads is good. We need a correct address to send an ambulance.  Listen, I can’t tell the address. Across the Up-river Bento, around Breeze Xin-Yi .  Help me to check.  In front of the Hotel.  Right in front of the Hotel.  In front of the Hotel, right?  Zhong-Xiao East Road. Zhong-Xiao East Road.  Is he, the victim, is he awake? Is he breathing normally?  He’s lying still.  He’s lying on the ground. Dose he move?  He’s twitching  He’s twitching  That traffic lights. I suggest you send an ambulance.  Yes, ambulance is dispatched. Is it male or female?  How old? What vehicles are involved?  Male, female, female. Female, female.  Female. What vehicles are involved?  Motorcycle and motorcycle collision.  OK, the ambulance.  I suggest you hurry.  Hold on. Don’t panic. OK, our ambulance is on the road.  Just lying on the ground, motionless.  Don’t touch him if he can’t move on his own.  Right at the traffic light here.  The traffic light at the Zhong-Xiao East Road.  Across the hotel. I suggest that the ambulance coming faster.  The ambulance is dispatched. Don’t panic.  Ok. Right.  Ok. Thanks. |
| --- |
