# Supplementary material for: Machine Learning–Based Text Analysis to Predict Severely Injured Patients in Emergency Medical Dispatch: Model Development and Validation
Source: J Med Internet Res. 2022 Jun 10;24(6):e30210. doi: 10.2196/30210 (PMC9233260; doi:10.2196/30210)
Supplement: Multimedia Appendix 2 [file jmir_v24i6e30210_app2.docx]

Appendix 2. Equations and Python script used in the model.

- Term frequency-inverse document frequency (TF-IDF)

TF-IDF is composed of two parts, TF and IDF, as can be seen in Equation (1). Here, ${tf}_{ij}$ is the occurrence of word *i* in document j, ${idf}_{i}$ is the logarithmically scaled inverse fraction of documents that contain word *i, N* is the total number of documents in the entire corpus, and ${df}_{i}$ is the number of documents containing word *i*.

|  | $w_{ij}= {tf}_{ij} \cdot{idf}_{i}$ | (1) |
| --- | --- | --- |
|  | ${idf}_{i}= log\frac{N}{{df}_{i}}$ | (2) |

- Bernoulli naïve Bayes (BNB)

In Equation (3), $V$ is the vocabulary set selected by the TF-IDF feature selection. $w_{t}$ indicates the $t$^th^ vocabulary of document *D*. $b_{t}$ is a Boolean expression indicating the presence or absence of the corresponding $w_{t}$ in the document, which forms the feature vector $\boldsymbol{b}$ for document *D*. Due to the assumption of conditional independence of each vocabulary, the likelihood of a document given by class $C_{k}$, $P\left( D | C_{k} \right),$ can be presented by this formula. The maximum a posteriori (MAP) determines the document category as can be seen in Equation (4). MAP of each class $(C_{k})$ will be calculated to illustrate the optimal estimation of the document distribution and the feature vector $\boldsymbol{b}$ that can perfectly explain the document *D*. The category with higher MAP owns the label of the document.

|  | $P\left( D \vert C_{k} \right)= P\left( \boldsymbol{b} \vert C_{k} \right)=\prod_{t=1}^{\vert V\vert} [b_{t}P\left( w_{t} \vert C_{k} \right)+\left( 1-b_{t} \right) (1-P\left( w_{t} \vert C_{k} \right))]$ | (3) |
| --- | --- | --- |

|  | $P\left( \boldsymbol{b} \vert C_{k} \right) P\left( C_{k} \right) \propto P(C_{k})\prod_{t=1}^{\vert V\vert} [b_{t}P\left( w_{t} \vert C_{k} \right)+\left( 1-b_{t} \right) (1-P\left( w_{t} \vert C_{k} \right))]$ | (4) |
| --- | --- | --- |

- **Python script of the PAMT model**

import numpy as np

import pandas as pd

import random

from sklearn.model_selection import train_test_split

from sklearn.feature_extraction.text import TfidfVectorizer

from sklearn.naive_bayes import BernoulliNB

from sklearn import metrics

from sklearn.metrics import confusion_matrix

# synonyms grouping

def synonyms(text):

combine_dict = {}

for line in open("synonyms.txt", "r", encoding='utf-8'): # input pre-built synonyms table

seperate_word = line.strip().split(" ")

for i in range(1, len(seperate_word)):

combine_dict[seperate_word[i]] = seperate_word[0]

new_text = []

for item in text:

new_text.append(item.split(' '))

final_text=[]

for item in new_text:

final = ''

for word in item:

if word in combine_dict:

word = combine_dict[word]

final += word + " "

else: final += word + " "

final_text.append(final)

return final_text

# rule-based judgment

def rule_based_judgment(test_out, y_pred):

t_group = []

for i in range(test_out.shape[0]):

t_incident = t_turnover = t_location = t_ppl = t_car = t_fly = t_react = t_stuck = t_lay = t_move = 0

for j in range(test_out.shape[1]-1, test_out.shape[1]-2, -1): # 連環車禍

if test_out[i][j] > 0:

t_incident += 1

for j in range(test_out.shape[1]-2, test_out.shape[1]-3, -1): # 翻覆

if test_out[i][j] > 0:

t_turnover += 1

for j in range(test_out.shape[1]-3, test_out.shape[1]-4, -1): # 國道

if test_out[i][j] > 0:

t_location += 1

for j in range(test_out.shape[1]-4, test_out.shape[1]-6, -1): # 老人家、行人

if test_out[i][j] > 0:

t_ppl += 1

for j in range(test_out.shape[1]-6, test_out.shape[1]-8, -1): # 公車、聯結車

if test_out[i][j] > 0:

t_car += 1

for j in range(test_out.shape[1]-8, test_out.shape[1]-10, -1): # 嚴重、飛出去

if test_out[i][j] > 0:

t_fly += 1

for j in range(test_out.shape[1]-10, test_out.shape[1]-13, -1): # 昏迷、沒有反應、不能講話

if test_out[i][j] > 0:

t_react += 1

for j in range(test_out.shape[1]-13, test_out.shape[1]-18, -1): # 有受困、夾住、卡住、車底、裡面

if test_out[i][j] > 0:

t_stuck += 1

for j in range(test_out.shape[1]-18, test_out.shape[1]-22, -1): # 倒地、躺著、爬不起來、壓住

if test_out[i][j] > 0:

t_lay += 1

for j in range(test_out.shape[1]-22, test_out.shape[1]-24, -1): # 不動、抽動

if test_out[i][j] > 0:

t_move += 1

t_total = [t_incident, t_turnover, t_location, t_ppl, t_car, t_fly, t_react, t_stuck, t_lay, t_move]

t_group = t_total

for t in range(len(t_total)):

if t_total[t] >= 1:

t_group[t] = 1

if (sum(t_group) >= 2) and (sum(t_total) >= 2): # at least 2 PAMT keywords in a file

y_pred[i] = 1 # change the prediction of the file to PAMT

return y_pred

# evaluation metrics calculation

def metric_calculation(BNB_conf_mat):

BNB_sensitivity = BNB_conf_mat[1][1] / (BNB_conf_mat[1][1] + BNB_conf_mat[1][0])

BNB_specificity = BNB_conf_mat[0][0] / (BNB_conf_mat[0][0] + BNB_conf_mat[0][1])

if BNB_conf_mat[1][1] != 0:

BNB_PPV = BNB_conf_mat[1][1] / (BNB_conf_mat[1][1] + BNB_conf_mat[0][1])

else:

BNB_PPV = 0

BNB_NPV = BNB_conf_mat[0][0] / (BNB_conf_mat[0][0] + BNB_conf_mat[1][0])

BNB_accuracy = (BNB_conf_mat[0][0] + BNB_conf_mat[1][1]) / (BNB_conf_mat[0][0] + BNB_conf_mat[1][1] + BNB_conf_mat[0][1] + BNB_conf_mat[1][0])

return BNB_sensitivity, BNB_specificity, BNB_PPV, BNB_NPV, BNB_accuracy

def main():

random_times = 100 # bootstrapping

keyword_num = 160 # the number of keywords

randomlist = [random.randint(0,100) for i in range(random_times)] # create random seed for bootstrapping

BNB_all_sensitivity = []

BNB_all_specificity = []

BNB_all_PPV = []

BNB_all_NPV = []

BNB_all_accuracy = []

for seed in randomlist:

### Input Segmented text ###

g_df = pd.read_csv('Segmented_text_non_PAMT.csv', index_col=0) # Non-PAMT texts

t_df = pd.read_csv('Segmented_text_PAMT.csv', index_col=0) # PAMT texts

g_label = pd.DataFrame([0 for i in range(72)], columns=['label']) # labels of Non-PAMT

t_label = pd.DataFrame([1 for i in range(42)], columns=['label']) # labels of PAMT

### Data Splitting ###

g_X_train, g_X_test, g_y_train, g_y_test = train_test_split(g_df, g_label, test_size=0.09, random_state = seed) # Non-PAMT : 65 training data + 7 testing data

t_X_train, t_X_test, t_y_train, t_y_test = train_test_split(t_df, t_label, test_size=0.06, random_state = seed) # PAMT: 39 training data + 3 testing data

### Synonyms grouping ###

X_train = synonyms(g_X_train['sentence'].tolist()) + synonyms(t_X_train['sentence'].tolist()) # Training data

X_test = synonyms(g_X_test['sentence'].tolist()) + synonyms(t_X_test['sentence'].tolist()) # Testing data

y_train = g_y_train['label'].tolist() + t_y_train['label'].tolist()

y_test = g_y_test['label'].tolist() + t_y_test['label'].tolist()

### Feature Engineering ###

# Feature Extraction

all_vectorizer = TfidfVectorizer('X_train', max_df = 0.95, min_df = 2, norm='l2', max_features = keyword_num)

all_vectorizer.fit_transform(X_train)

words = all_vectorizer.get_feature_names() # keywords per trial

# Feature addition of 37 words suggested by the dispatchers

suggested_word = ['意識','呼吸','清醒','自摔',

'站得起來','沒有受困','在動','可以講話',

'小車禍',

'自行就醫',

'皮肉傷','擦傷',

'巷子','巷弄',

'不動','抽動',

'倒地','躺著','爬不起來','壓住',

'有受困','夾住','卡住','車底','裡面',

'昏迷','沒有反應','不能講話',

'嚴重','飛出去',

'公車','聯結車',

'老人家','行人',

'國道',

'翻覆',

'連環車禍'

]

for i in suggested_word:

if i not in words:

words.append(i)

else:

words.remove(i)

words.append(i)

# Feature Extraction after combining the suggested features

train_vectorizer = TfidfVectorizer('X_train', vocabulary = words)

test_vectorizer = TfidfVectorizer('X_test', vocabulary = words)

train = train_vectorizer.fit_transform(X_train)

test = test_vectorizer.fit_transform(X_test)

train_out = train.toarray()

test_out = test.toarray()

### Classification ###

# BNB classifier

BNB = BernoulliNB()

BNB.fit(train_out, y_train)

BNB_y_pred = BNB.predict(test_out)

# Rule-based judgment #

BNB_y_pred = rule_based_judgment(test_out, BNB_y_pred)

### Evaluation ###

BNB_conf_mat = confusion_matrix(y_test, BNB_y_pred)

BNB_sensitivity, BNB_specificity, BNB_PPV, BNB_NPV, BNB_accuracy = metric_calculation(BNB_conf_mat)

BNB_all_sensitivity.append(BNB_sensitivity)

BNB_all_specificity.append(BNB_specificity)

BNB_all_PPV.append(BNB_PPV)

BNB_all_NPV.append(BNB_NPV)

BNB_all_accuracy.append(BNB_accuracy)

### Overall evaluation score ###

BNB_mean_sensitivity = round(np.mean(BNB_all_sensitivity, axis=0),3)

BNB_mean_specificity = round(np.mean(BNB_all_specificity, axis=0),3)

BNB_mean_PPV = round(np.mean(BNB_all_PPV, axis=0),3)

BNB_mean_NPV = round(np.mean(BNB_all_NPV, axis=0),3)

BNB_mean_accuracy = round(np.mean(BNB_all_accuracy, axis=0),3)

if __name__ == '__main__':

main()
