# Supplementary material for: Machine Learning–Based Text Analysis to Predict Severely Injured Patients in Emergency Medical Dispatch: Model Development and Validation
Source: J Med Internet Res. 2022 Jun 10;24(6):e30210. doi: 10.2196/30210 (PMC9233260; doi:10.2196/30210)
Supplement: Multimedia Appendix 3 [file jmir_v24i6e30210_app3.docx]

| Appendix 3. Comparison of machine learning models |
| --- |

No hyperparameters tuning is required for the MNB and BNB. The category estimation of the document for these two models depends on the maximum a posteriori (MAP) of each class, which consists of the likelihood of a document given by each class and its prior probability. Moreover, to avoid a zero-probability situation, Laplace smoothing was used to set the additive smoothing parameter to one.

The cluster k of KNN is set to 2 due to the two categories of the trauma severity. The MLP with the best results has a hidden layer with 25 neurons. Rectified Linear Unit (ReLU) activation and Adam optimizer are used. Moreover, the learning rate is set to 0.01 with 0.0001 l2 regularization, and the maximum number of iterations is 80.

The hyperparameter tuning of the remaining models, decision tree and SVM, adopts the grid search approach. In 100 times of RRS-CV (Repeated Random Subsampling-Cross Validation), the best parameter set selected for each validation is through 3-fold cross validation. For decision tree, maximum depth are the most influential parameters for the classification results of this study. Therefore, the model can choose the range of the maximum depth is from 1 to 4. The other parameter settings referred to the reference^*^. As can be seen in Figure A, the combination with higher accuracy but divergent distribution is when the maximum depth equals to one. As for SVM, the classification results are sensitive to the hyperparameters of the regularization parameter (C), kernel, and gamma when the kernel is rbf and sigmoid. The various parameter sets are based on three different kernels, linear, rbf, sigmoid, and the regularization parameter which ranges from 0.01 to 30. The other hyperparameters are set according to the reference^*^. Figure B shows the model performance of the three kernels with different gamma and regularization parameters. Different parameter combinations yield different levels of performance; however, these combinations achieve better classification results for each experiment in RRS-CVs for not only decision trees but SVM.


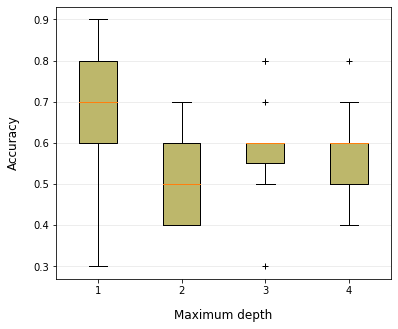


Figure A. Accuracy of Decision Tree at 4 different maximum depth values


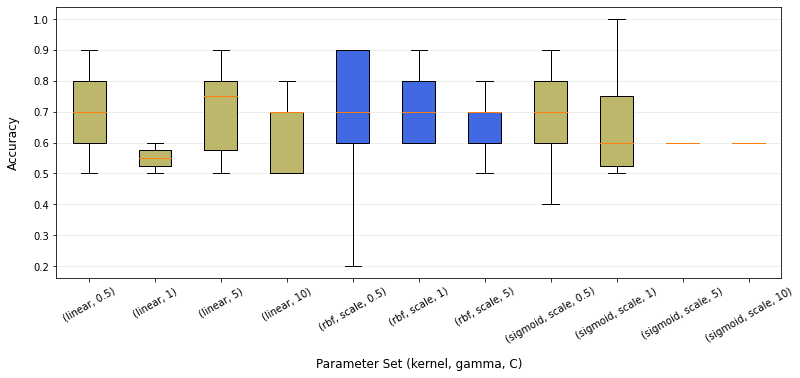


Figure B. Accuracy of SVM across 11 parameter sets

^*^Reference:

L. Buitinck *et al.*, “API design for machine learning software: experiences from the scikit-learn project,” Accessed: Dec. 28, 2021. [Online]. Available: https://github.com/scikit-learn.
