# Supplementary material for: Machine Learning–Based Text Analysis to Predict Severely Injured Patients in Emergency Medical Dispatch: Model Development and Validation
Source: J Med Internet Res. 2022 Jun 10;24(6):e30210. doi: 10.2196/30210 (PMC9233260; doi:10.2196/30210)
Supplement: Multimedia Appendix 4 [file jmir_v24i6e30210_app4.docx]

| Appendix 4. Descriptive statistics for audio and text files | | | | |
| --- | --- | --- | --- | --- |
|  | | Non-PAMT  (n = 72) | PAMT^b^  (n = 42) | Total  (n = 114) |
| Audio length^a^ (seconds) | | | | |
|  | Minimum | 24 | 26 | 24 |
|  | Maximum | 145 | 128 | 145 |
|  | Mean | 56 | 64 | 58.9 |
|  | SD^b^ | 24 | 24 | 24.5 |
| Text length (character count) | | | | |
|  | Minimum | 84 | 95 | 84 |
|  | Maximum | 652 | 516 | 652 |
|  | Mean | 227 | 266 | 241.4 |
|  | SD | 107 | 102 | 106.7 |
| ^a^Audio: audio files are generated from the original complete text content through text-to-speech tools.  ^b^Abbreviation: PAMT, prehospital activated major trauma; SD, standard deviation. | | | | |
