# Supplementary material for: Machine Learning–Based Text Analysis to Predict Severely Injured Patients in Emergency Medical Dispatch: Model Development and Validation
Source: J Med Internet Res. 2022 Jun 10;24(6):e30210. doi: 10.2196/30210 (PMC9233260; doi:10.2196/30210)
Supplement: Multimedia Appendix 5 [file jmir_v24i6e30210_app5.docx]

Appendix 5. Profiles and predictive performances of the participating dispatchers.

| Participant | Sex | Age (years), range | Service city | EMT^a^ experience (year) | Dispatch experience (year) | SENS^b^ (%) | SPEC^c^ (%) | PPV^d^ (%) | NPV^e^ (%) | ACC^f^ (%) |
| --- | --- | --- | --- | --- | --- | --- | --- | --- | --- | --- |
| A | Male | 30-39 | New Taipei City | 13 | 6 | 45.2 | 90.3 | 73.1 | 73.9 | 73.4 |
| B | Female | 40-49 | New Taipei City | 10 | 2 | 52.4 | 90.3 | 75.9 | 76.5 | 76.3 |
| C | Male | 30-39 | New Taipei City | 14 | 1 | 73.8 | 76.4 | 64.6 | 83.3 | 75.4 |
| D | Male | 30-39 | New Taipei City | 10 | 1 | 73.8 | 80.6 | 68.9 | 84.1 | 78.1 |
| E | Male | 30-39 | Taipei City | 10 | 4 | 54.8 | 88.9 | 74.2 | 77.1 | 76.3 |
| F | Male | 30-39 | Taipei City | 9 | 4 | 78.6 | 83.3 | 73.3 | 87.0 | 80.9 |
| Mean(SD^g^) | -- | -- | -- | -- | -- | 63.1 (13.9) | 85 (5.8) | 71.7 (4.2) | 80.3 (5.2) | 76.8 (2.5) |

^a^EMT: emergency medicine technician.

^b^SENS: sensitivity.

^c^SPEC: specificity.

^d^PPV: positive predictive value.

^e^NPV: negative predictive value.

^f^ACC: accuracy.

^g^SD: standard deviation.
