# Supplementary material for: Machine Learning–Based Text Analysis to Predict Severely Injured Patients in Emergency Medical Dispatch: Model Development and Validation
Source: J Med Internet Res. 2022 Jun 10;24(6):e30210. doi: 10.2196/30210 (PMC9233260; doi:10.2196/30210)
Supplement: Multimedia Appendix 6 [file jmir_v24i6e30210_app6.docx]

Appendix 6. Feature correlation analysis

In the study, among the 100 times of repeated random subsampling-cross validation (RRS-CV), 98 keywords are always selected as the feature set. These 98 critical features were used to implement the feature correlation analysis. Since emergency calls are often short and semantically intermittent, important words might appear frequently. Therefore, we emphasize on the correlation about how often words appear simultaneously and separately.

Phi coefficient is used to measure binary correlation. The measurement is shown in Table 1 and Equation (1). $n_{11}$ indicates the number of both word X and word Y appear in the same call. $n_{00}$ shows the number of times that none of them appear. $n_{10}$ and $n_{01}$ respectively refer to the number of documents when only word X or word Y appears. $n_{1\cdot}$ is the total number of documents that contains word X; $n_{0\cdot}$ is the total number of documents that does not contain word X. The number of files containing and not containing the word Y are denoted by $n_{\cdot1}$ and $n_{\cdot0}$ respectively. The coefficient ranges from -1 to 1. The larger the value, the higher the co-occurrence of the two words. 0 means that the variables appear randomly.

Table 1 Variables of Phi coefficient

|  | word Y appears | No word Y | Total |
| --- | --- | --- | --- |
| word X appears | $n_{11}$ | $n_{10}$ | $n_{1\cdot}$ |
| No word X | $n_{01}$ | $n_{00}$ | $n_{0\cdot}$ |
| Total | $n_{\cdot1}$ | $n_{\cdot0}$ | n |

Phi coefficient:

|  | $\emptyset= \frac{n_{11}n_{00}- n_{10}n_{01}}{\sqrt{n_{1\cdot}n_{0\cdot}n_{\cdot0}n_{\cdot1}}}$ | (1) |
| --- | --- | --- |

Figure 1 depicts the correlation of the top 30 keywords ranked by term frequency–inverse document frequency (TF-IDF). These 30 features are the most representative keywords of road accident emergency calls. The specific correlation relationship is shown in Table 2. Words with high correlation mean that they often co-occur or not co-occur in documents. These features are words that often appear together in Mandarin or information that needs to be asked in an emergency call. In contrast, words with low correlation mean that they often appear separately in documents. When the correlation coefficient equals to 0, the two features are randomly appear in documents without specific patterns. Regardless of the correlation between the word pairs in the table, in this study, each of these words is the most representative word in road accident emergency calls. Every word is meaningful, independent, and may be a potential keyword that is able to help in practical applications.


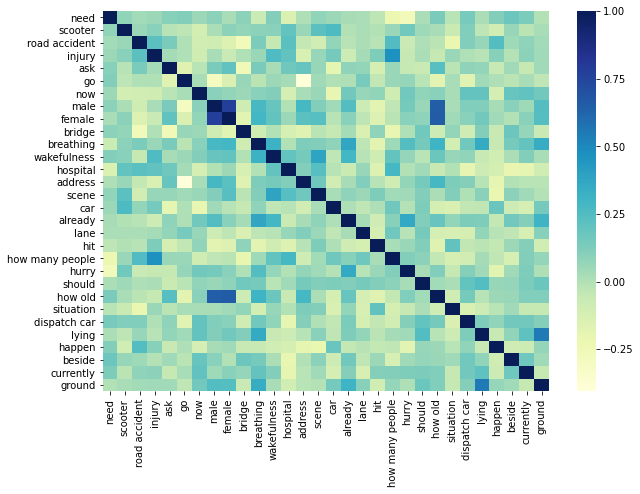


Figure 1 Feature correlation of the top 30 keywords ranked by TF-IDF

Table 2 Correlation of top 30 features selected by TF-IDF

| Correlation relationship | 1^st^ feature | 2^nd^ feature | Coefficient |
| --- | --- | --- | --- |
| High correlation | Male | Female | 0.780 |
|  | Female | How old | 0.655 |
|  | Male | How old | 0.653 |
|  | Lying | Ground | 0.551 |
| No correlation | Breathing | Lying | 0.000 |
|  | Male | Hospital | 0.000 |
|  | Breathing | Hospital | 0.000 |
|  | Hurry | Hospital | 0.000 |
|  | Situation | Hospital | 0.000 |
|  | Female | Beside | 0.000 |
| Low correlation | Go | Address | -0.406 |
