# Supplementary material for: Machine Learning–Based Text Analysis to Predict Severely Injured Patients in Emergency Medical Dispatch: Model Development and Validation
Source: J Med Internet Res. 2022 Jun 10;24(6):e30210. doi: 10.2196/30210 (PMC9233260; doi:10.2196/30210)
Supplement: Multimedia Appendix 7 [file jmir_v24i6e30210_app7.docx]

| Appendix 7. Relation of the length of text and accuracy. | | | | | | |
| --- | --- | --- | --- | --- | --- | --- |
| Length of text | Word count | Case number | | | Accuracy (%) | |
|  |  | Non-PAMT | PAMT | Total | PAMT model | Participants |
| Shorter text^a^ | ≤165 | 21 | 8 | 29 | 79.3 | 78.2 |
| Longer text^b^ | ≥308 | 14 | 15 | 29 | 72.4 | 75.9 |
| ^a^ Shorter text: calls with the 25% fewest words.  ^b^ Longer text: calls with the 25% most frequent words.  Abbreviation: PAMT, prehospital activated major trauma  The results of the relationship between the text length and the classification accuracy are shown here. We found that in terms of the dispatchers and the PAMT model, the accuracy of shorter texts is slightly higher than that of longer texts. However, each text underwent a text preprocessing step and is represented by features based on the word frequency, this relationship between the original length and accuracy of the text needs further research to justify it. | | | | | | |
