# Supplementary material for: Machine Learning–Based Text Analysis to Predict Severely Injured Patients in Emergency Medical Dispatch: Model Development and Validation
Source: J Med Internet Res. 2022 Jun 10;24(6):e30210. doi: 10.2196/30210 (PMC9233260; doi:10.2196/30210)
Supplement: Multimedia Appendix 8 [file jmir_v24i6e30210_app8.docx]

| Appendix 8. Representative keywords for PAMT chosen by experts and PAMT model | | | | |
| --- | --- | --- | --- | --- |
| Category | Expert (23 words) | | PAMT model (top 23 words) | |
| Patient status | 車底^a^ | Under the vehicle^a^ |  | |
|  | 有受困^a^ | Trapped^a^ |  |  |
|  | 抽動^a^ | Twitching^a^ |  |  |
|  | 嚴重^a^ | Severe^a^ |  |  |
|  | 不動^b^ | Motionless, still^b^ | 嚴重^a^ | Severe^a^ |
|  | 壓住^b^ | Pinned under^b^ | 抽動^a^ | Twitching^a^ |
|  | 沒有反應^b^ | No response^b^ | 有受困^a^ | Trapped^a^ |
|  | 夾住^b^ | Pinched, trapped^b^ | 車底^a^ | Under the vehicle^a^ |
|  | 倒地^b^ | Fall off^b^ | 沒有受困 | Not trapped |
|  | 躺著^b^ | Lying^b^ | 在動 | Moving |
|  | 爬不起來^b^ | Can’t get up^b^ |  | |
|  | 裡面^b^ | Inside^b^ |  |  |
|  | 昏迷^b^ | Coma^b^ |  |  |
|  | 不能講話 | Can’t speak |  |  |
|  | 卡住 | Stuck |  |  |
| Patient basic information | 老人家^b^ | Elderly^b^ | 客人 | Guest |
|  | 行人^b^ | Pedestrian^b^ |  |  |
| Mechanism | 公車^a^ | Bus^a^ | 公車^a^ | Bus^a^ |
|  | 飛出去 | Fly away/off/out, ejected |  |  |
|  | 翻覆 | Overturned | 被撞 | Being bumped |
|  | 聯結車 | Combination vehicle |  |  |
|  | 連環車禍 | Pileup car crash |  |  |
| Geographic information | 國道^a^ | Freeway, highway^a^ | 國道^a^ | Freeway, highway^a^ |
|  |  |  | 高架 | Overpass |
|  |  |  | 成功路 | Cheng-Gong Rd. |
|  |  |  | 加油站 | Gas station |
|  |  |  | 仁愛 | Ren-Ai (name of a road) |
|  |  |  | 士林 | Shi-Lin (name of an area) |
| Auxiliary words and other information | -- | | 看看 | Check |
|  |  |  | 出來 | Get out |
|  |  |  | 紅綠燈 | Traffic light |
|  |  |  | 公里 | Kilometer |
|  |  |  | 不要急 | No hurry/rush |
|  |  |  | 看不出來 | Cannot tell |
|  |  |  | 看起來 | Seem/look like |
|  |  |  | 剛好 | Just |

^a^Overlapped words of the left and right columns.

^b^Overlapped words of the left column and all words extracted through term frequency–inverse document frequency.

Abbreviation: PAMT, prehospital activated major trauma
